# Case Report: Therapeutic plasma exchange for systemic centipede envenomation in a 2-kg Chihuahua dog

**DOI:** 10.3389/fvets.2026.1900868

**Published:** 2026-09-29

**Authors:** Wei Tang, Ye Li, Guoyang Sun, Chenyiyu Zheng, Zhaoxi Gao, Shuai Li

**Affiliations:** 1 Shanghai Veterinary Research Institute, Chinese Academy of Agricultural Sciences, Shanghai, China; 2 Shanghai AnAn Pengfeng Pet Hospital, New Ruipeng Pet Healthcare Group Co., Ltd, Shanghai, China; 3 Shanghai GlinX Biotechnology Co., Ltd, Changning, Shanghai, China

**Keywords:** centipede envenomation, Chihuahua, extracorporeal blood purification, therapeutic plasma exchange, toy-breed dog

## Abstract

A 2-kg, 8-year-old neutered male Chihuahua was presented after a centipede sting to the interdigital region of the right forepaw, followed by lameness and red urine. Wound care and intravenous fluid therapy were provided at the referring clinic; however, pigmenturia persisted and systemic manifestations developed, prompting referral. On admission, the dog had icteric mucous membranes, mild dehydration, and clinicopathologic evidence of systemic inflammation, tissue injury, hepatocellular damage, and suspected hemolysis. Because of the acute systemic manifestations following envenomation and persistence of clinical abnormalities despite initial medical management, membrane-based therapeutic plasma exchange (TPE) was performed. Estimated plasma volume was 96 mL, and the prescribed exchange volume was 134 mL (1.4 plasma volumes). Approximately 1,080 mL of whole blood was processed over 3 h. The 68-mL extracorporeal circuit was primed with whole blood, anticoagulation was achieved with heparin, and fresh frozen canine plasma was used as the replacement fluid. TPE was completed over 3 h at a whole blood flow rate of 6 mL/min; transient asymptomatic ionized hypocalcemia was detected and treated with calcium gluconate. After treatment, C-reactive protein decreased from 8.9 to 1.5 mg/dL and appetite returned, although depression initially persisted. The dog subsequently improved during hospitalization and was discharged after 7 days. In this dog, membrane-based TPE was well tolerated despite the small body size and was temporally associated with improvement in clinical signs.

## 1 Introduction

Therapeutic plasma exchange (TPE) is an extracorporeal blood purification technique in which plasma containing pathogenic substances is removed and replaced with a physiologically compatible fluid, such as albumin solutions, plasma products, crystalloids, or synthetic colloids. In human medicine, TPE is widely used for selected intoxications, autoimmune diseases, and severe inflammatory conditions (1). In veterinary medicine, TPE has increasingly been applied in dogs for immune-mediated diseases, including immune-mediated hemolytic anemia, myasthenia gravis and intoxications such as non-steroidal anti-inflammatory drug overdose (2–4).

Centipede venom is secreted from the venom glands associated with the forcipules and contains a complex mixture of bioactive substances, including neurotoxins, proteolytic enzymes, histamine, and serotonin (5). Envenomation can result in local pain and swelling, but systemic manifestations such as hemolysis, inflammation, tissue injury, and, in severe cases, multi-organ dysfunction have also been described (6). Conventional treatment is generally supportive and may include wound care, analgesia, antihistamines, and fluid therapy. However, these measures may be insufficient once venom components and secondary inflammatory mediators have entered the circulation.

Here, we describe the successful use of TPE in a 2-kg Chihuahua with systemic centipede envenomation. To the best of our knowledge, this is the first reported canine case in which TPE was used for this indication.

## 2 Case description

### 2.1 History and presenting complaints

An 8-year-old neutered male Chihuahua weighing 2 kg was referred for evaluation after stepping on a centipede during an evening walk. The centipede species was not identified. An interdigital lesion characterized by erythema and a suspected hemorrhagic focus was visible on the right forepaw, and the dog showed pain and lameness. Approximately 1 h later, the owner observed passage of red urine, and the dog was taken to a local clinic. The wound was disinfected, and vitamin C and intravenous lactated Ringer’s solution were administered. Because the red urine persisted, the dog was referred to our hospital approximately 7 h after envenomation.

### 2.2 Clinical findings

At presentation, the dog was depressed, had poor appetite, and showed cranial abdominal pain on palpation. Body temperature was 38.9 °C, respiratory rate was 14 breaths/min, heart rate was 110 bpm, and systolic blood pressure was 120 mmHg. Capillary refill time was 1–2 s, dehydration was estimated at 5%, and the mucous membranes were icteric. Erythema was present in the right inguinal region, without visible petechiae or ecchymoses. Although the dog was no longer lame at the time of referral, the interdigital lesion on the right forepaw remained visible.

### 2.3 Diagnostic assessment

The dog was DEA 1 negative. Hematologic analysis revealed leukocytosis, neutrophilia, and an increased neutrophil percentage, consistent with systemic inflammation. Blood smear evaluation showed platelet aggregation, neutrophilia, and a left shift, supporting toxic inflammatory change. Ghost cells and spherocytes were not observed, and a saline agglutination test was negative. Serum biochemistry demonstrated marked increases in creatine kinase (CK), lactate (LAC), C-reactive protein (CRP), pancreatic lipase (PL), alanine aminotransferase (ALT), and total bilirubin (TBili) indicating muscle injury, metabolic stress, systemic inflammation, hepatocellular injury, possible pancreatic injury, and hyperbilirubinemia.

Before heparin administration, coagulation testing revealed a prolonged activated partial thromboplastin time (aPTT) of 158 s (reference interval, 60–93 s), whereas PT was within the reference interval at 14 s (reference interval, 11–14 s). Blood gas analysis of a venous sample showed decreased PCO2 and base excess in extracellular fluid (BEecf). The principal abnormal hematologic, biochemical, and blood gas findings are summarized in Table 1.

**Table 1:** Selected clinicopathologic results obtained before therapeutic plasma exchange.

Parameter	Result	Reference interval	Unit	Flag
WBC	20.03	5.32–16.92	10^9^/L	↑
Neu	17.49	3.05–12.10	10^9^/L	↑
Neu%	87.3	42.0–84.0	%	↑
PCO2	25.0	35.00–38.00	mmHg	↓
PO2	38	35–50	mmHg	
BEecf	−7	−5.00–0.00	mmol/L	↓
ALT	290	10–125	U/L	↑
CK	575	10–200	U/L	↑
LAC	3.50	0.50–2.50	mmol/L	↑
PL	1,459	0.0–200	U/L	↑
CRP	8.9	0.0–1.0	mg/dL	↑
aPTT	158	60–93	s	↑
PT	14	11–14	s	
TBili	44	0–15	μmol/L	↑
HCT	42.4	35.0–60.0	%	

Urinalysis was performed on a sample collected by ultrasound-guided cystocentesis. The urine was red and turbid, with a specific gravity of 1.016 and a pH of 8.0. The dipstick was strongly positive for blood/heme (250 Ery/μL), with bilirubin at 1 mg/dL and urobilinogen at 4 mg/dL, whereas microscopic examination of the urine sediment showed no red blood cells. These findings supported pigmenturia with suspected hemoglobinuria and intravascular hemolysis, although myoglobinuria could not be definitively excluded.

To exclude common infectious causes of hemolytic disease, a six-pathogen real-time quantitative PCR (qPCR) panel was performed using the InCycle automated nucleic acid detection system and a six-plex canine vector-borne pathogen nucleic acid detection kit (Shanghai GlinX Biotechnology Co., Ltd., Shanghai, China). Results were negative for *Ehrlichia* spp., *Babesia gibsoni*, *Babesia canis*, hemotropic *Mycoplasma* spp., *Hepatozoon* spp., and *Anaplasma phagocytophilum*.

Based on the history of recent centipede sting, the acute onset of clinical signs, and the clinicopathologic findings, the dog was diagnosed with systemic centipede envenomation complicated by suspected hemolysis, acute liver injury, and systemic inflammation.

## 3 Therapeutic intervention

After clinical assessment and discussion with the owner, TPE was initiated approximately 20 h after envenomation because of persistent pigmenturia and systemic clinical abnormalities despite initial medical management.

Membrane-based TPE was performed using a Plasauto Σ blood purification system (Asahi Kasei Medical Co., Ltd., Japan) with an OP-02W plasma separator. A 5-Fr, 13-cm double-lumen catheter was aseptically placed in the right external jugular vein to establish vascular access. The tubing set had a priming volume of 43 mL and the plasma separator had a volume of 25 mL, resulting in a total extracorporeal circuit volume of 68 mL. Total blood volume (TBV) was estimated at 160 mL using 80 mL/kg; thus, the extracorporeal circuit represented approximately 42.5% of the estimated TBV. The measured pre-TPE hematocrit (HCT) was 42.4% and was approximated to 40% for the prescription. Plasma volume (PV) was calculated as TBV × (1–0.40), yielding an estimated PV of 96 mL. Because the prescribed exchange dose for TPE is commonly 1.0–1.5 plasma volumes, the prescribed exchange volume for this dog was set at 1.4 PV, corresponding to approximately 134 mL, and the full prescribed volume was exchanged (Figure 1).

**Figure 1:**
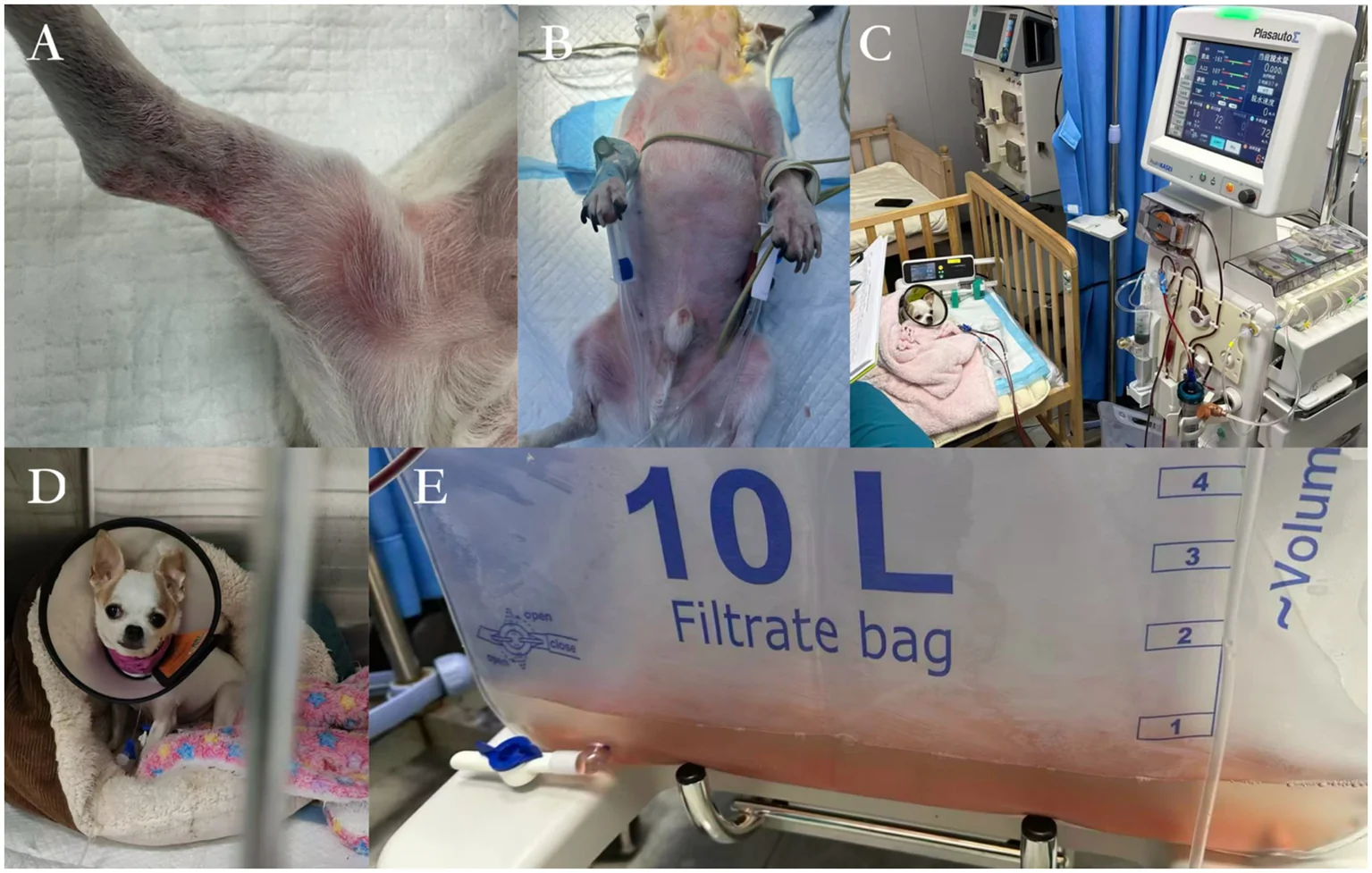
Clinical findings, therapeutic plasma exchange procedure, and post-treatment appearance in a dog with centipede envenomation. **(A)** Erythema in the right inguinal region at hospital admission. **(B)** Multifocal generalized erythema that developed immediately after propofol administration. **(C)** The dog undergoing therapeutic plasma exchange (TPE). **(D)** Clinical appearance of the dog after TPE. **(E)** Plasma discarded at the end of TPE, showing marked reddish discoloration.

During preparation for TPE, immediately after propofol administration, multifocal erythema developed over the ventral abdomen and thorax, axillae, and ventral neck, and an acute hypersensitivity reaction was suspected. Dexamethasone (1 mg, IV) and diphenhydramine (4 mg, IM) were administered for treatment. The extracorporeal circuit was primed with 68 mL of whole blood. Fresh frozen canine plasma (134 mL) was used as the replacement fluid and infused at a rate and volume matched to plasma removal. The replacement fluid was maintained at 40 °C. The whole blood flow rate was set at 6 mL/min, and the target plasma separation ratio of 20% was achieved. Using the standard hematocrit-based calculation, the plasma separation rate was 6 × (1–0.40) × 0.20 × 60 = 43.2 mL/h; no metabolic correction factor was applied. The prescribed and actual plasma exchange volumes were both 134 mL. TPE was completed in approximately 3 h. Based on the prescribed blood flow rate and treatment duration, approximately 1,080 mL of whole blood, equivalent to approximately 6.8 estimated total blood volumes, was processed during the session. Unfractionated heparin (62.5 IU, IV) was administered before TPE, followed by a constant-rate infusion of 31.25 IU/kg/h. The target aPTT range was 150–200 s. Serial aPTT values were 208, 198, 179, and 170 s at 0.5, 1, 1.5, and 3 h, respectively. Because anticoagulation was considered adequate and no circuit clotting or increase in transmembrane pressure occurred, the heparin infusion rate was not adjusted. No extracorporeal circuit clotting occurred, and transmembrane pressure remained stable at 10–15 mmHg throughout the procedure.

During TPE, body temperature was maintained with a warming blanket, and electrolytes and aPTT were monitored periodically. Before TPE, ionized calcium, total calcium, and potassium concentrations were 1.21 mmol/L, 2.29 mmol/L, and 3.8 mmol/L, respectively. Approximately 1 h after TPE was initiated, ionized calcium decreased to 0.77 mmol/L, while potassium was 3.7 mmol/L. A constant-rate infusion of 10% calcium gluconate was then administered at 1 mL/h for approximately 2 h until completion of TPE (total dose, approximately 2 mL); ionized calcium was 1.02 mmol/L after treatment. No clinical signs of hypocalcemia were observed. At the end of the procedure, the discarded plasma showed marked reddish discoloration. A total of 40 mL of commercially available 5% human albumin was used as a rinse-back fluid to return approximately 40 mL of blood remaining in the extracorporeal circuit. The partial restitution volume was selected on the basis of clinical judgment, taking into account the estimated 5% dehydration at admission, blood loss associated with serial sampling during TPE, and anticipated minor blood loss due to clot retention within the extracorporeal circuit. The remaining circuit contents were discarded. No clinical evidence of volume overload was observed. Doppler systolic blood pressure remained between 110 and 130 mmHg, and no hypotension or hypersensitivity reaction occurred during TPE.

Following TPE, the dog received lactated Ringer’s solution by constant-rate infusion at 4 mL/h for 3 days. Potassium chloride was supplemented intravenously according to electrolyte monitoring to correct hypokalemia. Additional supportive medications included vitamin K1 (10 mg, SC), compound vitamin B (1 mL, IV) for 3 days, amoxicillin-clavulanate (50 mg, PO, BID) for 5 days, ulinastatin (100,000 IU [50,000 IU/kg], IV, SID) for 7 days, and prednisolone (1 mg/dog/day [0.5 mg/kg/day], PO) for 5 days.

## 4 Outcome and follow-up

Clinicopathologic variables were serially monitored after TPE. Immediately after treatment, CRP decreased from 8.9 mg/dL to 1.5 mg/dL, representing an 83% reduction. A transient rebound occurred during the first 2 days after TPE, after which CRP continued to decline and returned to the reference interval (Figure 2A). A second TPE session was not performed because the dog continued to improve clinically and CRP subsequently declined. Pancreatic lipase decreased from 1,459 U/L before TPE to 883 U/L after treatment, representing a 40% reduction. On day 3, PL increased again to 1716 U/L and subsequently declined to 345 U/L (Figure 2B).

**Figure 2:**
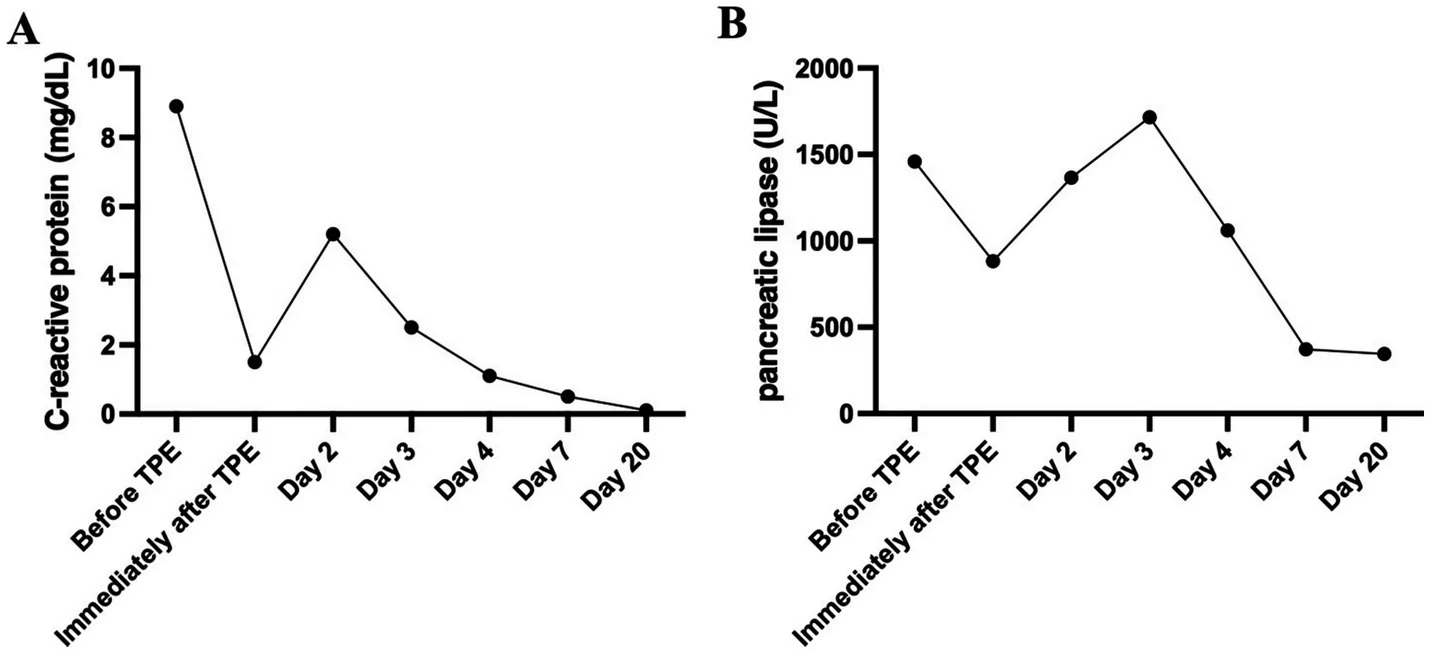
Serial changes in C-reactive protein and pancreatic lipase concentrations before and after therapeutic plasma exchange (TPE). **(A)** Serial changes in C-reactive protein concentration. **(B)** Serial changes in pancreatic lipase concentration.

Other abnormal biochemical variables also improved during hospitalization. ALT decreased from 290 to 83 U/L, CK decreased from 575 to 150 U/L, and lactate decreased from 3.50 to 0.70 mmol/L, all returning to within their respective reference intervals. Total bilirubin decreased from 44 μmol/L on the day of envenomation to 3.4 μmol/L immediately after TPE. Grossly red urine was no longer observed after TPE. Because urinalysis and urine sediment examination were not repeated after treatment, complete resolution of hemolysis could not be confirmed. Serial erythrogram measurements are summarized in Table 2.

**Table 2:** Serial erythrogram measurements before and after therapeutic plasma exchange.

Variable	Day of envenomation	Before TPE	1 day after TPE	6 days after TPE
HGB (g/L)	151	139	154	154
RBC (×10^12^/L)	6.74	6.18	6.80	6.84
HCT (%)	46.4	42.4	47.1	48.6

Following TPE, appetite returned, although the dog remained depressed initially. Mentation subsequently improved during hospitalization, and the icterus gradually resolved. No abnormalities were detected on physical examination at discharge 7 days after admission. At the 20-day recheck, the owner reported normal appetite, activity, and behavior. Physical examination was unremarkable, and inflammatory and hepatic indicators had normalized. Pancreatic lipase remained mildly increased but continued to trend downward (Figure 3).

**Figure 3:**
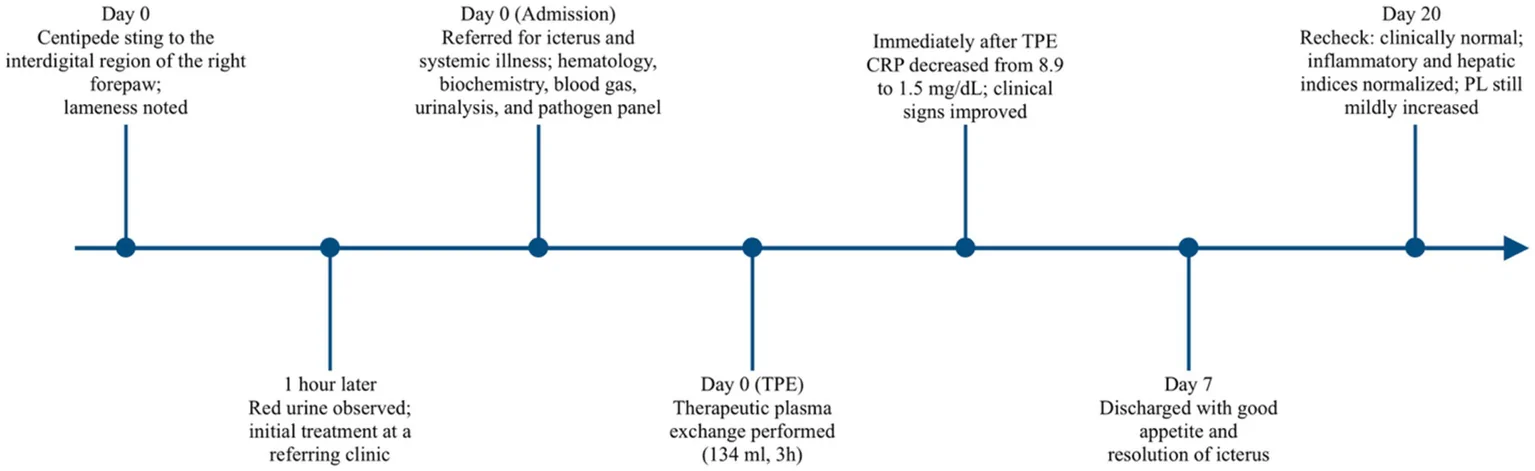
Timeline of clinical presentation, therapeutic plasma exchange, and follow-up in a 2-kg Chihuahua with centipede envenomation.

## 5 Discussion

This case highlights two clinically important points. First, TPE was technically feasible and well tolerated in an extremely small dog weighing only 2 kg. Second, TPE was temporally associated with subsequent clinical and biochemical improvement in a dog with systemic centipede envenomation.

Performing TPE in toy-breed dogs is challenging because of their limited blood volume, small vessel size, and lower physiologic reserve. In this dog, the 68-mL extracorporeal circuit represented approximately 42.5% of the estimated total blood volume. For comparison, a previously reported canine membrane-based TPE system had a circuit volume of 71 mL and was used in dogs with a median body weight of 13.0 kg (range, 3.3–45.0 kg) (7). The whole blood flow rate used here (6 mL/min) was substantially lower than the rates commonly reported in larger dogs (7, 8). Such a low flow rate prolongs blood transit through the extracorporeal circuit and may increase the risk of circuit clotting during membrane-based TPE. This risk is particularly relevant in a 2-kg dog because clotting of the circuit could result in loss of a clinically important proportion of the circulating blood volume. Blood priming, continuous heparin anticoagulation, and close monitoring of transmembrane pressure were therefore used. No extracorporeal circuit clotting occurred, and transmembrane pressure remained stable at 10–15 mmHg throughout the procedure.

TPE in a patient of this size also carries substantial risks of hypotension, hypothermia, electrolyte disturbances, bleeding, transfusion-associated reactions, and extracorporeal circuit blood loss (9). The large extracorporeal-to-circulating blood volume ratio increases the potential for hemodynamic instability, while prolonged extracorporeal circulation may predispose very small patients to hypothermia. In this case, active warming was provided and the replacement plasma was maintained at 40 °C. Doppler systolic blood pressure remained between 110 and 130 mmHg, and no clinically apparent hypotension or volume overload occurred during the procedure. Transient ionized hypocalcemia was the principal treatment-related complication observed. Ionized calcium decreased from 1.21 mmol/L before TPE to 0.77 mmol/L approximately 1 h after initiation of treatment. Although heparin rather than citrate was used for circuit anticoagulation, citrate contained in the fresh frozen plasma replacement fluid could still chelate ionized calcium and likely contributed to this decrease (10). A constant-rate infusion of 10% calcium gluconate was initiated, after which ionized calcium increased to 1.02 mmol/L by the end of TPE. No clinical signs attributable to hypocalcemia were observed. These findings indicate that membrane-based TPE was technically achievable in this 2-kg dog, but the procedure should not be considered low risk and requires close hemodynamic, thermal, coagulation, and electrolyte monitoring.

The generalized erythema observed in this dog developed immediately after propofol administration and before initiation of TPE; therefore, dexamethasone and diphenhydramine were administered to treat a suspected acute hypersensitivity reaction. Although plasma transfusion reactions are possible in dogs, a prospective multicenter study reported acute reactions in 4.5% of plasma-product transfusions (11). No additional hypersensitivity reaction occurred during TPE in this dog.

The mechanisms of injury following centipede envenomation are likely multifactorial. Centipede venom contains a complex mixture of inflammatory and cytotoxic components that may cause both local tissue injury (12) and systemic inflammatory responses (13). Because the centipede species was not identified in this case and venom composition varies among species, findings from individual species cannot be directly extrapolated to this dog. In this dog, the presence of icterus, pigmenturia, increased inflammatory markers, hyperlactatemia, elevated CK activity, hepatocellular enzyme leakage, and prolonged aPTT before heparin administration supported a diagnosis of clinically significant systemic envenomation. A factor Xa-inhibiting peptide isolated from *Scolopendra subspinipes mutilans* venom has been shown experimentally to prolong coagulation times, providing biologic plausibility for venom-associated coagulation abnormalities but not establishing the cause of the prolonged aPTT in this dog (14). Conventional treatment generally relies on local analgesia, antihistamines, and supportive care, but these measures do not directly eliminate circulating venom components, providing a rationale for considering TPE in patients with severe systemic manifestations (15). In the present case, TPE may have been beneficial by removing circulating venom-associated components, pigment-containing plasma, bilirubin, and inflammatory mediators (16, 17). However, the clinical manifestations and natural course of centipede envenomation in dogs remain poorly characterized, with very limited veterinary literature available (18). Consequently, there is insufficient evidence to determine clear indications for TPE in canine centipede envenomation. In this case, the decision to perform TPE was based on the combination of persistent pigmenturia and multiple systemic clinicopathologic abnormalities, but its necessity cannot be definitively established.

The marked immediate decreases in CRP and total bilirubin after TPE should be interpreted cautiously because TPE directly removes circulating plasma solutes. In a canine membrane-based TPE series, serum bilirubin decreased by a median of 36% during treatment (7), indicating that an immediate post-exchange decrease may partly reflect physical removal and dilution rather than resolution of the underlying disease process. The subsequent increase in CRP during the first 2 days may have reflected redistribution, continued hepatic synthesis, or the expected kinetics of an evolving acute-phase response; canine CRP can continue to increase during the first 24–48 h after an acute inflammatory insult (19). A second TPE session was not performed because the dog continued to improve clinically and CRP subsequently declined.

Persistent pancreatic lipase elevation during follow-up warrants cautious interpretation. Although pancreatic injury could not be excluded, pancreatic lipase should not be interpreted in isolation or considered diagnostic of pancreatitis without compatible clinical findings and supportive imaging (20). In this case, the dog remained clinically improved and the value declined over time, suggesting either resolving pancreatic injury or nonspecific secondary pancreatic involvement associated with systemic inflammation. Continued dietary management and follow-up monitoring were therefore recommended.

The principal limitation of this report is that the effect of TPE cannot be separated from that of concurrent treatments. In addition to fluid and supportive therapy, the dog received corticosteroids, diphenhydramine, amoxicillin-clavulanate, and ulinastatin, all of which may have influenced clinical recovery or inflammatory markers. Experimental evidence in dogs suggests that ulinastatin can modify physiologic and inflammatory responses in septic shock, although evidence for its efficacy in canine centipede envenomation is lacking (21, 22). Other limitations include the single-case design, unknown centipede species, absence of venom measurements, lack of repeat urinalysis and urine sediment examination after TPE, and direct removal of measured plasma biomarkers by the procedure itself. Consequently, the temporal association between TPE and improvement does not establish causality. Because the dog was hemodynamically stable at presentation and severe canine centipede envenomation is poorly characterized, whether TPE was necessary to achieve recovery cannot be determined from this case. The primary contribution of this report may therefore be the demonstration of the technical feasibility and risks of membrane-based TPE in an extremely small dog, rather than proof of efficacy for centipede envenomation.

In conclusion, membrane-based TPE was technically feasible and well tolerated in this 2-kg dog and was temporally associated with subsequent clinical and clinicopathologic improvement. However, the efficacy and indications for TPE in canine centipede envenomation remain uncertain. Additional case series and comparative studies are needed to determine efficacy, optimal timing, and treatment protocols.

## Data Availability

The raw data supporting the conclusions of this article will be made available by the authors, without undue reservation.
